# Effects of probiotics and antibiotics on the intestinal homeostasis in a computer controlled model of the large intestine

**DOI:** 10.1186/1471-2180-12-47

**Published:** 2012-03-27

**Authors:** Ateequr Rehman, Femke-Anouska Heinsen, Marjorie E Koenen, Koen Venema, Henrik Knecht, Stephan Hellmig, Stefan Schreiber, Stephan J Ott

**Affiliations:** 1 Department of Environmental Health Sciences, University Medical Center, Breisacher Str. 115b, D-79106 Freiburg, Germany; 2 Institute for Clinical Molecular Biology (ICMB), Christian-Albrechts-University (CAU) Kiel, Schittenhelmstr. 12, D-24105 Kiel, Germany; 3 TNO, Utrechtseweg 48, P.O. Box 360, 3700 AJ Zeist, The Netherlands; 4 Department of Internal Medicine I, University Hospital Schleswig-Holstein (UKSH), Campus Kiel, Arnold-Heller Str. 3, Haus 6, D-24105 Kiel, Germany; 5 Institute for Clinical Molecular Biology (IKMB), Christian-Albrechts University (CAU) Kiel and Clinic for Internal Medicine I University-Hospital, Schleswig-Holstein (UK S-H), Kiel Arnold-Heller-Str. 3, Haus 6, 24105 Kiel, Germany

## Abstract

**Background:**

Antibiotic associated diarrhea and *Clostridium difficile *infection are frequent complications of broad spectrum antibiotic therapy. Probiotic bacteria are used as therapeutic and preventive agents in these disorders, but the exact functional mechanisms and the mode of action are poorly understood. The effects of clindamycin and the probiotic mixture VSL#3 (containing the 8 bacterial strains *Streptococcus thermophilus*, *Bifidobacterium breve*, *Bifidobacterium longum*, *Bifidobacterium infantis*, *Lactobacillus acidophilus*, *Lactobacillus plantarum*, *Lactobacillus paracasei *and *Lactobacillus delbrueckii *subsp. *Bulgaricus*) consecutively or in combination were investigated and compared to controls without therapy using a standardized human fecal microbiota in a computer-controlled *in vitro *model of large intestine. Microbial metabolites (short chain fatty acids, lactate, branched chain fatty acids, and ammonia) and the intestinal microbiota were analyzed.

**Results:**

Compared to controls and combination therapy, short chain fatty acids and lactate, but also ammonia and branched chain fatty acids, were increased under probiotic therapy. The metabolic pattern under combined therapy with antibiotics and probiotics had the most beneficial and consistent effect on intestinal metabolic profiles. The intestinal microbiota showed a decrease in several indigenous bacterial groups under antibiotic therapy, there was no significant recovery of these groups when the antibiotic therapy was followed by administration of probiotics. Simultaneous application of anti- and probiotics had a stabilizing effect on the intestinal microbiota with increased bifidobacteria and lactobacilli.

**Conclusions:**

Administration of VSL#3 parallel with the clindamycin therapy had a beneficial and stabilizing effect on the intestinal metabolic homeostasis by decreasing toxic metabolites and protecting the endogenic microbiota from destruction. Probiotics could be a reasonable strategy in prevention of antibiotic associated disturbances of the intestinal homeostasis and disorders.

## Background

Antibiotic-associated diarrhea (AAD) and *Clostridium difficile *infection (CDI) are frequent complications of broad-spectrum antibiotic therapy. In a large prospective multicenter study, AAD was observed in 4.9% of the patients (1.8%-6.9%) receiving long-term antibiotic treatment with > 50% of patients showing positive testing for *C. difficile *toxin B [1]. The incidence of CDI is still increasing [2,3] and the disease is complicated by the occurrence of virulent and pathogenic *C. difficile *ribotypes associated with higher morbidity and mortality, which are responsible for CDI outbreaks worldwide [4]. The increasing incidence and mortality associated with the CDI and the significant rate of treatment failures and recurrences with current antibiotics emphasize the role of preventative strategies.

Probiotics are promising agents in the prevention of AAD and CDI. Originally they were used in the therapy of AAD and CDI and for regeneration of intestinal microbiota after antibiotic treatment. A significant reduction of AAD and CDI could be observed in randomized clinical trials when administered simultaneously with the antibiotic substances [5-8]. Probiotic microbes have positive impact on microbe-microbe and host-microbe interactions, and could also limit pathogen by modulating gut microbiome competitive interactions and/or by producing antimicrobial compounds [9-11]. Reports state positive effect of probiotics on beneficial short chain fatty acid production and negative on harmful net ammonia production [12,13].

However, the heterogeneity of probiotic formulations and the vague definition of probiotics as otherwise not classified microorganisms that improve health of the host impede the assessment of clinical trials. Several effects have been attributed to probiotics, among them direct influences on the composition of intestinal microbiota, the intestinal metabolism and the immune response [14-16], but the exact mode of action is poorly understood. Previously, we have developed a validated, dynamic *in vitro *model of the gastrointestinal tract [17], which allows for mode of action studies to be performed.

Mechanistic studies are difficult to perform *in vivo *due to difficulties in sampling and ethical considerations. The *in vitro *gastrointestinal model of the colon simulates to a high degree the successive dynamic processes in the large intestine [17]. The model is a unique tool to study the stability, release, dissolution, absorption and bioconversion of nutrients, chemicals, bioactive compounds and pharmaceuticals in the gastrointestinal tract [18,19]. Besides the average physiological conditions and the biological variation, also abnormal or specific conditions can be simulated in a reproducible way. The following standardized conditions are simulated: body temperature; pH in the lumen; delivery of a pre-digested substrate from the 'ileum'; mixing and transport of the intestinal contents; presence of a complex, high density, metabolically active, anaerobic microbiota of human origin; and absorption of water and metabolic products via a semipermeable membrane inside the colon model [17]. This model has been validated successfully with regards to the number and ratio of the various micro-organisms which are similar in composition and metabolic activity with that of the human colon. Furthermore, it has been validated for the production of metabolites, such as short-chain fatty acids (SCFA), branched-chain fatty acids (BCFA), gases, ammonia, and phenolic compounds and used for studies on bioconversion of flavonoids [18] or glucosinolates by the human colon microbiota [19].

The *in vitro *system can support scientific research, e.g. studying the role of specific micro-organisms in the fermentation of dietary fibers, the fate and function of probiotics and other foods or drugs, and the development of novel products in a shorter time.

In this study different therapeutic regimens (antibiotics; probiotics following antibiotics; antibiotics and probiotics together; no therapy) were investigated in the *in vitro *model using a standardized intestinal human microbiota originating from healthy adult volunteers. We monitored beneficial (SCFA and lactate) and putrefactive/toxic (BCFA and ammonia) metabolites. The intestinal microbiota composition was also analyzed under the different conditions.

## Methods

### Test products

The two test products were Clindamycin and VSL #3. Clindamycin (Fresenius Kabi, Bad Homburg, Germany) is a broad-spectrum lincosamide antibiotic usually used to treat anaerobic infections. It is effective against most Gram-positive cocci and Gram-negative anaerobic bacteria and comparable with macrolide antibiotics. VSL#3 (Sigma-tau, Duesseldorf, Germany) is a multi-species probiotic and contains the following 8 species: *Streptococcus thermophilus*, *Bifidobacterium breve*, *Bifidobacterium longum*, *Bifidobacterium infantis*, *Lactobacillus acidophilus*, *Lactobacillus plantarum*, *Lactobacillus paracasei *and *Lactobacillus delbrueckii *subsp. *bulgaricus*.

### Ethical approval

A general ethical committee vote for the collection of stool samples of healthy volunteers had been obtained from the local ethical board of the Medical Faculty of the Christian-Albrechts-University (CAU) in Kiel. All volunteers have given informed consent.

Test system: TNO large-intestinal model (TIM-2)

**Figure 1:**
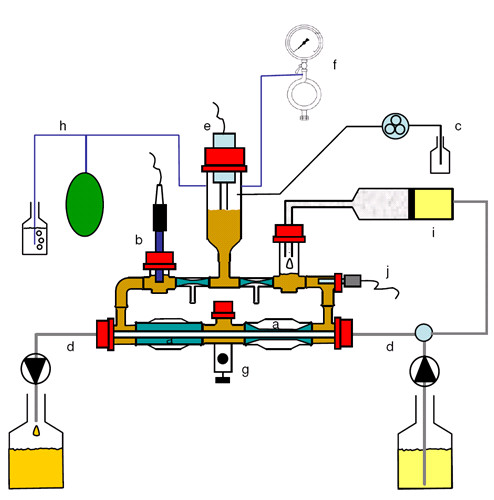
**Schematic representation of the TNO TIM-2 in vitro model with (a) peristaltic compartments containing fecal matter; (b) pH electrode; (c) alkali pump; (d) dialysis liquid circuit with hollow fibre membrane; (e) level sensor; (f) N_2 _gas inlet; (g) sampling port; (h) gas outlet; (i) 'ileal efflux' container containing SIEM; (j) temperature sensor**. In brief, the model consists of four glass units with a flexible wall inside (peristaltic compartments) and a total volume of 135 ml. Water of body temperature (37°C) was pumped into the space between the glass jacket and the flexible wall, causing the microbiota to be mixed and moved. The sequential squeezing of the walls, controlled by a computer, caused a peristaltic wave forcing the material to circulate through the loop-shaped system. Physiological electrolyte and metabolite concentrations in the lumen were maintained with a dialysis system consisting of hollow fibres, running through the lumen of the reactor, through which dialysis liquid was pumped at a speed of 1.5 ml/min. The model further contained an inlet system for delivery of the artificial ileal delivery medium (SIEM), and a level sensor to maintain the luminal content at the set level of 135 ml. The system was kept anaerobic by flushing with gaseous nitrogen. At the start of each experiment the model was inoculated with 30 ml of the standard, cultivated faecal microbiota, consisting of a mix of fecal samples from 7 individuals. The composition of this microbiota consisted of all microbes present in the fecal donations (unpublished data).

The study was performed in the TNO dynamic system of the large intestine (TIM-2) as schematically represented in Figure 1 and as described in detail by Venema et al. [20] and Minekus et al. [17].

### Microbiota

The study was performed in TIM-2 with an active microbiota originating from ten healthy adults. Inclusion and exclusion criteria were: age between 20 and 70 years, no chronic or active disease, no medication (including any antibiotic or pre/probiotic treatment at least 6 weeks prior to enrolment in the study), no pregnancy, and no stay at hospital within the last 6 months. The mean age was 46.3 years, the gender ratio m:f was 5:5. Stool samples were collected and immediately snap-frozen in liquid nitrogen at -196°C. The material was shipped on dry ice to TNO. In order to increase the reproducibility of the inoculation a standardized microbiota was prepared from these stools according to Venema et al. [20].

### Micro-ecological studies

After inoculation of the system with the microbiota the experiments started with a 16 hour stabilization period in which the microbiota could adapt to the system. Thereafter the test period started. In the control unit the standard ileal efflux meal (SIEM) was fed to the system. SIEM was given at a rate of 56 ml/day. Its composition is described in Maathuis et al. (2009). In brief, it contained the following components: 2.5 g K_2_HPO_4_.3H_2_O, 4.5 g NaCl, 0.005 g FeSO_4_.7H_2_O, 0.5 g MgSO_4_.7H_2_O, 0.45 g CaCl_2_.2H_2_O, 0.4 g cysteine.HCl, 4.7 pectin, 4.7 xylan, 4.7 arabinogalactan, 4.7 amylopectin, 23.5 casein, 39.2 starch, 17 Tween 80, 23.5 bactopeptone, 0.4 bile, plus 1 ml of a vitamin mixture containing (per litre): 1 mg menadione, 2 mg D-biotin, 0.5 mg vitamin B12, 10 mg pantothenate, 5 mg nicotinamide, 5 mg p-aminobenzoic acid and 4 mg thiamine. The pH was kept constant at 5.8. The antibiotic was administered as a shot at the start of the experiment (1.5 mg) and furthermore the antibiotic was administered with the SIEM (0.75 mg/day) and it was added to the dialysate (10 mg/l) in order to prevent dialysis of antibiotic out of the lumen. Dialysis liquid contained (per litre): 2.5 g K_2_HPO_4_.3H_2_O, 4.5 g NaCl, 0.005 g FeSO_4_.7H_2_O, 0.5 g MgSO_4_.7H_2_O, 0.45 g CaCl_2_.2H_2_O, 0.4 g cysteine.HCl, 0.05 bile, plus 1 ml of the vitamin mixture. The probiotic compound was administered at a dose of 4.4 g per day containing at least 450 billion bacteria (according to the manufacturer), and was administered as a single shot each 24 h after dissolving the powder is 10 ml dialysis liquid.

**Figure 2:**
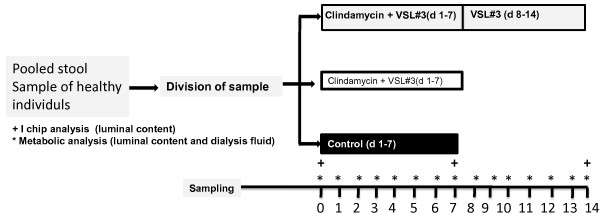
**Schematic representation of study design and mode of sampling**. A pooled stool sample was assigned to the three study arms (Clindamycin for 7 days followed by VSL#3 for 7 days, Clindamycin + VSL#3 for 7 days, no therapy control for 7 days). Dialysis fluid and lumen samples for metabolic analysis (SCFA, BCFA, lactate, ammonia) were collected daily, lumen samples for microbial analysis were sampled before therapy and at the end of each 7 days period.

In the TIM-2 experiments, the composition of the colon microbiota was followed in time after intake of the test compounds (Clindamycin and/or VSL#3) during several days at a frequent intervals (see Figure 2 for setup of the experiments). The control experiment without any addition was performed as a single run, the variation with the first 7 days addition of antibioics and then 7 days probiotics was performed in triplicate, while the variation with the combined addition of probiotics + antibiotics was performed in duplicate. Analysis of the composition of the microbiota indicated the bacterial genera which were selectively stimulated or suppressed by the test compounds. In addition, samples were analyzed for SCFA, BCFA, lactate and ammonia. These values provided an indication of the balance between health-promoting and toxic products produced by the microbiota after addition of the different compounds i) separately and consecutively or ii) in combination. Analysis of (changes in) these microbial metabolites provided information on the functionality of the changes that took place in the microbiota.

### Sampling

Before, during (every day at 24 h intervals) and at the end of the fermentation experiments, samples were taken from the lumen of the model and from the dialysis liquid for analysis on metabolites. Each day 25 ml was taken out of the system to simulate passage of stool. Additional samples were taken from the lumen of the colon model for analyzing the composition of the microbiota using the I-Chip platform (description later in this material and methods section). These samples were taken at day 0, day 7 and day 14.

### Short chain fatty acids (SFCA) and branched chain fatty acids (BCFA) analyses

The lumen and dialysis samples were analyzed gas-chromatographically on the concentrations of SCFA and BCFA as follows: Samples were centrifuged (12000 rpm, 5 min) and a mixture of formic acid (20%), methanol and 2-ethyl butyric acid (internal standard, 2 mg/mL in methanol) was added to the clear supernatant. According to the method described by Jouany [21] as described in detail by van Nuenen et al. [22], a 0.5-μL sample was injected on a GC-column (Stabilwax-DA, length 15 m, ID 0.53 mm, film thickness 0.1 mm; Varian Chrompack, Bergen op Zoom, The Netherlands) in a Chrompack CP9001 gas chromatograph using an automatic sampler (Chrompack liquid sampler CP9050; Varian Chrompack).

### Lactate

For lactate analysis the samples were centrifuged as described above. In the clear supernatant both L- and D-lactate were determined enzymatically (based on Boehringer, UV-method, Cat. No. 1112821) by a Cobas Mira plus autoanalyzer (Roche, Almere, The Netherlands), as described in detail by van Nuenen et al. [22]. The analysis is based on the conversion of NAD + into NADH.

### Ammonia

For the analysis for the protein-fermentative metabolite ammonia samples were centrifuged as described above and analyzed as described in detail by Van Nuenen et al. [22]. The analysis is based on the conversion of free ammonia with hypochlorite/phenol reagent into blue indophenol. In the clear supernatant indophenol was measured by measuring the absorbance at 600 nm with a Cobas Mira Plus autoanalyzer.

### I-Chip platform

The 'intestinal chip' (I-Chip) has been developed as a faster alternative method to determine the composition of the microbiota. Sequences of approximately 400 microorganisms have been placed on a DNA micro-array as previously described [23,24]. DNA was isolated from the luminal samples of the TIM-2 experiments. Subsequently the DNA was labeled and hybridized to DNA-arrays printed with the probes. After washing the arrays were scanned and analyzed. Analysis of the composition of the microbiota (using I-chip) indicated the bacterial genera which are selectively stimulated or suppressed by the antibiotic and/or probiotic. Changes in the composition of the microbiota in the experiments in which Clindamycin was applied for seven days, or in which Clindamycin plus probiotics were applied together for seven days, were compared with the changes in the control experiment in the same time period. Changes in the composition of the microbiota after application of probiotics sequentially after the application of Clindamycin were compared to the composition of the microbiota after the application of Clindamycin for seven days.

### SAM analysis

The data obtained with the I-chip were analyzed with Significance Analysis of Microarrays (SAM) for statistical relevance [25].

## Results and discussion

*In vivo*, Clindamycin shows good penetration into tissues and is often used to treat skin or soft tissue infections. Pseudomembranous colitis (PMC) caused by overgrowth of *Clostridium difficile *is a potentially life-threatening complication of antibiotic therapy. The probiotic product VSL#3 is a dietary supplement often used for treatment of various gastrointestinal complaints directly associated with microbial dysbiosis such as chronic constipation, diarrhea, flatulence, ulcerative colitis and pouchitis [16,26,27].

The *in vitro *model used in this study provides standardized and reliable conditions to study the effects of pro- and antibiotics on the human intestinal microbiota [17] and is has an advantage over living system in continuous sampling over a defined period of time. Moreover, the system is hardly biased by environmental factors, e.g. temperature, humidity or oxygen, which can be controlled to a high extent.

The TIM-2 experiments were performed using a standardized microbiota from healthy individuals. In the control unit the standard ileal efflux meal (SIEM) was fed to the system. In one experiment the antibiotic was administered together with a probiotic mixture (VSL#3) and in the other experiment the probiotic was administered after the antibiotic treatment.

### Production of beneficial microbial metabolites

**Figure 3:**
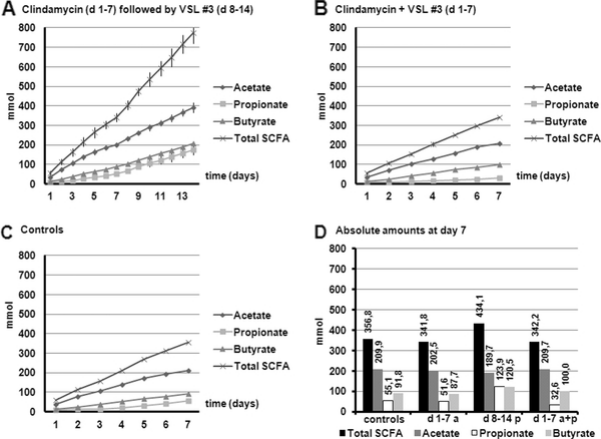
**Cumulative production of the short chain fatty acids (SCFA) acetate, propionate and n-butyrate during the different experiments in TIM-2: (A) Clindamycin for 7 days (d 1-7 a) followed by VSL#3 (d 8-14 p); (B) Clindamycin + VSL#3 for 7 days (d 1-7 a + p); (C) no therapy group for 7 days (controls)**. Figure 3D shows the comparison of absolute amounts (in mmol) at the end of each 7 days period.

Short chain fatty acids (SCFA) and lactate are beneficial microbial metabolites. SCFA and lactate acidify the intestinal lumen, causing growth arrest or even death of (opportunistic pathogens). In addition, the SCFA are an energy source for the host: butyrate for colonic epithelial cells, acetate and propionate, in amongst others liver and muscle cells [28-30]. Figure 3 presents cumulative total production of the short chain fatty acids, e.g acetate, propionate and n-butyrate during the different experiments in TIM-2, and represents metabolites present in lumen and dialysate. The amount of SCFA present at the start of the experiment has been artificially set to zero so the graphs only reflect the production of metabolites after start of addition of the test products.

The total SCFA production was not affected by the use of Clindamycin or Clindamycin plus probiotics. When probiotics were administered after the administration of Clindamycin for one week, the SCFA production increased since the slope of the total SCFA production increased in the second week, compared with the first week of the experiment. The production of n-butyrate and propionate was increased when probiotics were added. The acetate concentration was unaffected by the addition of Clindamycin or probiotics. When Clindamycin and probiotics were administered together the propionate production was decreased. These differences are likely to be caused by changes in the microbiota composition.

**Figure 4:**
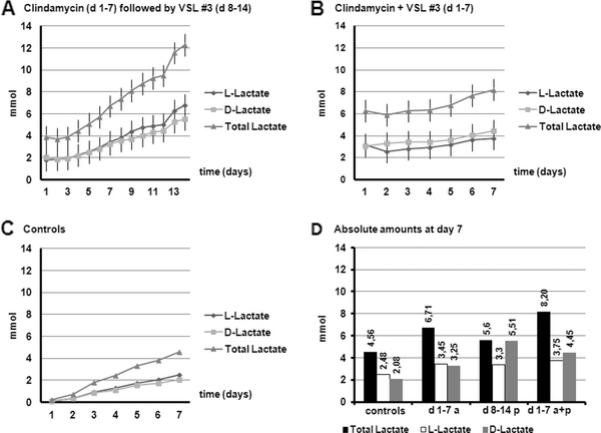
**Cumulative production of lactate (D- and L-lactate) during the different experiments in TIM-2: (A) Clindamycin for 7 days (d 1-7 a) followed by VSL#3 (d 8-14 p); (B) Clindamycin + VSL#3 for 7 days (d 1-7 a + p); (C) no therapy group for 7 days (controls)**. Figure 4D shows the comparison of absolute amounts (in mmol) at the end of each 7 days period.

Figure 4 presents the cumulative total production of lactate. Lactate was produced in all variations, but when probiotics were added the lactate production was increased, independent of the presence of Clindamycin. The probiotics were lactic acid bacteria and the extra production of lactate proved the probiotics were active in the microbiota. Lactate is only accumulating when there is a fast fermentation. If substrates are fermented slowly, lactate is converted into the other SCFA (primarily propionate and butyrate) and does not accumulate.

The total SCFA production was not affected by the use of antibiotics or antibiotics plus probiotics. When probiotics were added after using antibiotics, the SCFA production increased. Propionate production was decreased when antibiotics and probiotics were used together. Enhanced production of lactate was observed both when probiotics were administrated together with Clindamycin or when they were administered after seven days of clindamycin administration.

### Production of putrefactive microbial metabolites

Branched chain fatty acids (BCFA; iso-butyrate and iso-valerate) and ammonia are metabolites produced from protein fermentation, a process which is generally believed to be putrefactive and leading to production of toxic metabolites. These products are deleterious for host health [22].

**Figure 5:**
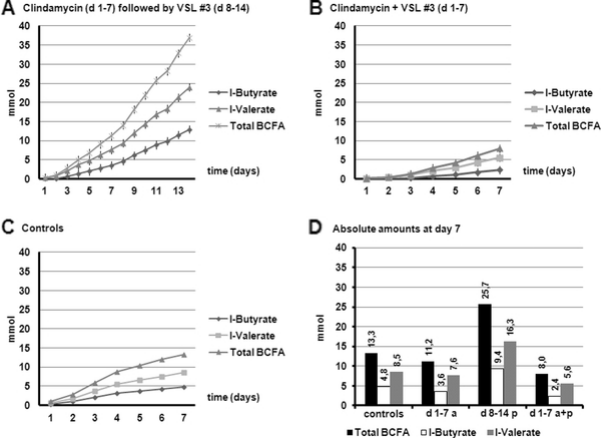
**Cumulative production for the branched chain fatty acids (BCFA) iso-butyrate and iso-valerate during the different experiments in TIM-2: (A) Clindamycin for 7 days (d 1-7 a) followed by VSL#3 (d 8-14 p); (B) Clindamycin + VSL#3 for 7 days (d 1-7 a + p); (C) no therapy group for 7 days (controls)**. Figure 5D shows the comparison of absolute amounts (in mmol) at the end of each 7 days period.

Figure 5 presents the cumulative total production of BCFA. BCFA are produced in small amounts for every test variation compared to the SCFA (about 20 to 40 fold lower). Total BCFA production was highest when probiotic was administered after clindamycin. However, when Clindamycin and probiotics were administered at the same time, the BCFA production was decreased. In the experiments in which Clindamycin was administered (the first 7 days), the BCFA production was comparable to the control. Therefore the decreasing effect probably was induced by the use of probiotics. When probiotics were administered after a week treatment with Clindamycin, this decreasing effect in BCFA production was not observed.

**Figure 6:**
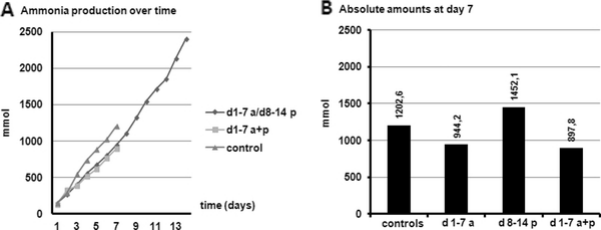
**Cumulative production for ammonia during the different experiments in TIM-2 (A) (Clindamycin for 7 days (d 1-7 a) followed by VSL#3 (d 8-14 p); Clindamycin + VSL#3 for 7 days (d 1-7 a + p); no therapy group for 7 days (controls)**. Figure 6B shows the comparison of absolute amounts (in mmol) at the end of each 7 days period.

Figure 6 shows the cumulative total production of ammonia. For ammonia the production was decreased between day 3 and 7 in the test experiments compared to the control. In the experiments in which Clindamycin was administered, as well as in which Clindamycin was administered together with probiotics, the ammonia production was reduced just as observed for the BCFA.

### Composition of the microbiota

To determine the effects of Clindamycin and the probiotics on the composition of the microbiota, the I-chip platform was used. The I-chip contained roughly 400 probes, some for group-level detection (e.g *Bifidobacterium *genus) and some for the detection of individual species (e.g. *Bifidobacterium longum*). Some groups and species were covered by more than one probe. In all cases the hybridization to these multiple probes correlated very well. However, not al probes gave a signal above background noise, which was expected, as not all microorganisms are present above the level of detection of the method (approximately 10^7 ^CFU/g). Due to the different nature of each probe (different sequence), hybridization intensity does not necessarily reflect abundance. Difference in GC-content results in different hybridization efficiencies. Although the I-Chip at most is semi-quantitative, comparing signals from one and the same hybridization does allow interpretation of the increase or decrease of certain probes. For the TIM-2 experiments samples from time points 0, 7 and 14 were analyzed.

**Figure 7:**
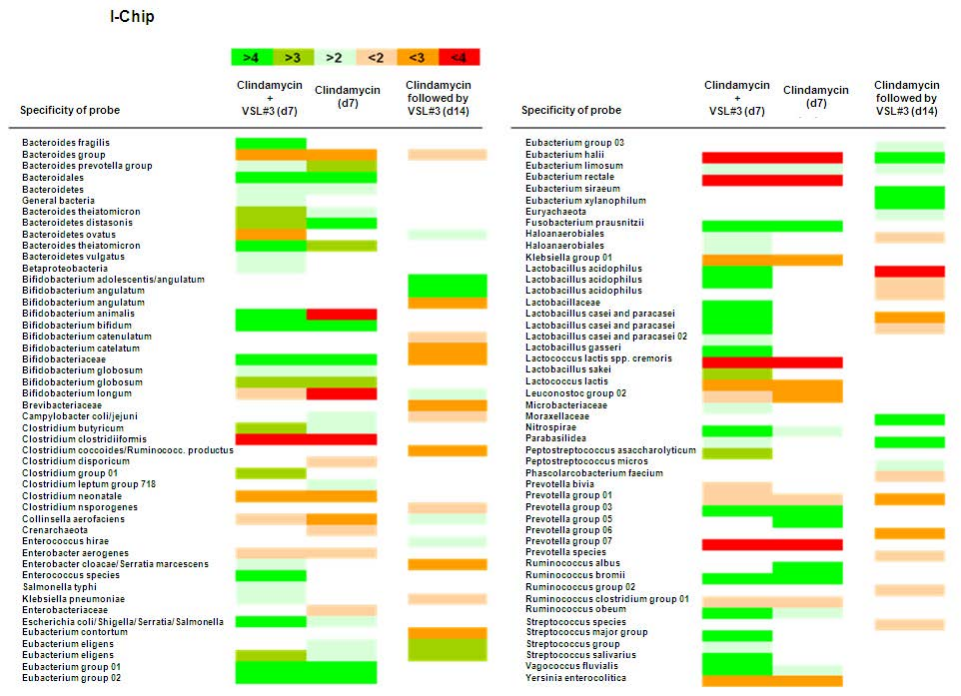
**Graphic representation of the I-chip results showing those probes that i) give a signal above the background, and ii) differed by a factor of > 2 from the control for the first two columns**. For the third column the effect of the addition of probiotics after treatment with Clindamycin was compared to the result after treatment with Clindamycin alone (middle column). Green signifies a factor of 2 or higher compared to the control (or antibiotic experiment at day 7) and red stands for a factor of 2 or more lower compared to the control (or antibiotic at day 7).

Figure 7 shows the results of the I-chip analysis. Displayed is the fold-increase in signal between the start and the end of the fermentation period compared to the control. For day 14 of the experiment with Clindamycin followed by probiotics the results at day 14 were compared with the same experiment at day 7, after Clindamycin only.

Different shades of green reflect more than 2, more than 3 and more than 4 times increases of microbial species, genera or groups compared to the control, while the different shades of red reflect the more than 2, 3 and 4 times decrease of microbial species, genera or groups compared to the control.

Comparing the experiments receiving Clindamycin to the control experiment, the experiments with administration of Clindamycin showed a decrease in Bifidobacerium animalis *Bifidobacterium longum*, Crenarchaeota, Enterobacteriaceae, *Lactococcus lactis *subsp. *cremoris*, *Lactococcus lactis *subsp. and an increase in *Bifidobacterium bifidum Eubacterium eligens, Bacteroidetes*, Bactetroidales*, Ruminococcus albus, Ruminococcus bromii *and *Fusobacterium prausnitzii*.

When Clindamycin and probiotics were administered together the following species increased compared to the control: *Bifidobacterium animalis*, *Enterobacter cloaca*/*Serratia marcesens*/*Salmonella typhi*, Enterococcus species, Haloanaerobiale, *Lactobacillus acidophilus*, Lactobacillaceae, *Lactobacillus casei *and *paracasei*, *Lactobacillus gasseri*, *Lactobacillus sakei*, Microbacteriaceae, Nitrospirae, *Parabasilidea peptostreptococcus asaccharolyticum*, Streptococcus groups and *Streptococcus salivarius*. *Bifidobacterium longum *(which was in the probiotic mixture) decreased less strong than when Clindamycin was administered alone.

When Clindamycin was administered for 7 days and the probiotics were administered the week thereafter the bacteria that increased compared to the situation after antibiotic treatment alone were *Bifidobacterium adolescentis*/*Bifidobacterium angulatum*, *Bifidobactrium longum*, Collinsella aerofaciens, *Enterococcus hirae*, *Eubacterium siraeum*, *Eubacterium xylanophilum*, Euryachaeota, Moraxellaceae and *Peptostreptococcus micros*. The groups that decreased were *Bifidobacterium catenulatum*, Bifidobacteriaceae, Brevibacteriaceae, *Campylobacter coli*/*jejuni*, *Clostridium coccoides*/*Ruminococcus productus*, *Clostridium sporogenes*, *Enterobacter cloacae*/*Serratia marcesens*, *Salmonella typhi*/*Klebsiella penumoniae*, *Eubacterium contortum*, Haloanaerobiales, *Lactobacillus acidophilus*, *Lactobacillus casei *and *paracasei *and *Phascolarcobacterium faecium*.

Administration of clindamycin together with probiotics has positive effect on lactobacilli while the administration of probiotic after antibiotic has negative effect on same bacterial group. For the bifidobacteria this seemed to be divided in two groups, increase in one group (namely *Bifidobacterium animalis*) was observed when Clindamycin together with probiotics, but not when probiotic was administated after Clindamycin. Decrease in another group (namely *Bifidobacterium catenulatum*) was observed only when probiotics were administrated after clindamycin but not in other experimental setups

Statistical analyses (SAM) of the data obtained with the I-chip showed that all time point 0 samples clustered together (data not shown) and thus could be considered equal. The SAM analysis did not add new information to the other analysis performed on the I-chip data.

According to the I-chip results not all strains from the probiotic mixture increased when the mix was added to the TIM-2 system; therefore we plated the mixture to get an idea of the amount and proportions of the bacterial strains in the mixture. The amount of bifidobacteria was very low in the mixture and only *Bifidobacterium longum *could be identified.

After administration of clindamycin, a decrease in bifidobacteria and lactococci groups was observed, whereas in the experiment in which Clindamycin was administered together with the probiotic mix, an increase in *Bifidobacterium animalis *as well as several *Lactobacillus *strains could be observed, and decrease of *Bifidobacterium longum *was also less strong, decreasing from 4 fold to 2 fold.

Increase in the beneficial bacterial group Lactobacilli was observed when Clindamycin and probiotics were administered together, while if the probiotics were administered following the administration of Clindamycin the level of lactobacilli was lower. In summary, in this study we could demonstrate that the simultaneous administration of anti- and probiotics had the most significant positive effects on intestinal homeostasis by stabilizing the intestinal microbial composition, increased production of short chain fatty acids and decreasing the production of toxic microbial metabolites like ammonia and other branched chain fatty acids. We could also show that probiotics are active when applied simultaneous with antibiotics. Therefore the administration of probiotics could be of significant advantage in the prevention of AAD and CDI by surveillance of intestinal metabolic balance.

## Conclusions

Administration of VSL#3 parallel with the clindamycin therapy had a beneficial and stabilizing effect on the intestinal metabolic homeostasis by decreasing toxic metabolites and protecting the endogenic microbiota from destruction. Probiotics could be a reasonable strategy in prevention of antibiotic associated disturbances of the intestinal homeostasis and disorders.

## Authors' contributions

AR participated in the design of the study and drafted the manuscript. FAH and HK performed basic experiments, participated in statistical analysis and helped preparing the graphs for the manuscript. MK and KV designed and performed the bioreactor experiments, they were involved in statistical analysis and preparing of graphs. SH and SS participated in the design of the study and sampling. SJO designed and coordinated the study, he prepared the manuscript and participated in the statistical analysis. All authors read and approved the final manuscript.
